# Behavior and Mechanism of High-Temperature Stability between Tial-Based Alloy and Y_2_O_3_-Al_2_O_3_ Composite Crucibles

**DOI:** 10.3390/ma11071107

**Published:** 2018-06-28

**Authors:** Qingling Li, Huarui Zhang, Yongshuang Cui, Chunlei Yang, Ming Gao, Jinpeng Li, Hu Zhang

**Affiliations:** 1 School of Materials Science and Engineering, Beihang University, Beijing 100191, China; qingling@buaa.edu.cn (Q.L.); gao_ming@buaa.edu.cn (M.G.); lijinpeng@buaa.edu.cn (J.L.); 2 China Aerospace Academy of Systems Science and Engineering, Beijing 100048, China; 15810535812@163.com; 3 Capital Aerospace Machinery Co., Ltd., Beijing 100076, China; yangchunlei985@163.com

**Keywords:** Y_2_O_3_, Al_2_O_3_, composite crucible, interface reaction, TiAl-based alloy, stability behavior

## Abstract

In this work, Y_2_O_3_ based composite crucibles with different Al_2_O_3_ contents were designed and characterized. The stability behaviors and interaction mechanisms between molten Ti-47Al-2Cr-2Nb alloy and Y_2_O_3_-Al_2_O_3_ composite crucibles were investigated at high temperature. Results showed that the surface morphology of crucibles and the degree of interfacial reactions between the composite crucibles and the metal melts varied with the change of Al_2_O_3_ content in the crucible matrix. The pure Y_2_O_3_ crucible was the densest and its chemical stability was the highest. With the increase in Al_2_O_3_ content, the number of pores on the crucibles surface gradually increased and the interfacial reactions between the composite crucibles and the molten alloys became weaker. When the content of Al_2_O_3_ in composite crucibles increased from 3.5 wt % to 10.5 wt %, the thickness of the interface layer of melt-crucible decreased from 150 µm to 50 µm, and the equilibrium contact angles between metal and crucibles gradually decreased from 69.3° to 64.2° at 1873 K.

## 1. Introduction

Intermetallic TiAl-based alloys with the advantages of low density, high specific strength and elastic modulus, excellent creep properties and high resistance to oxidation, have extensive application in aerospace, automotive and energy industries [1,2,3,4]. They are acknowledged to be the most promising candidates to replace Ni-based superalloys [5]. However, the applications of TiAl-based alloys are restricted due to low ductility and fracture toughness at low temperature [6]. Therefore, some methods must be developed for optimizing the comprehensive properties of TiAl-based alloys.

The melting process of an alloy is one of the most important factors affecting the properties of the alloy [7]. Many meaningful studies have been conducted to improve the quality of TiAl-based alloys. Researchers suggested that the molten TiAl-based alloys have high reactivity, which led to some interactions between the molten alloys and crucible materials during melting and casting, resulting in deterioration of the internal and external quality of castings [8]. Therefore, the selection of refractories during the process of melting is crucial for obtaining high-quality alloys.

Previous investigations have shown that oxide ceramic materials have a better and more extensive application, of which Al_2_O_3_, CaO, ZrO_2_ and Y_2_O_3_ are the most commonly used [9,10,11,12,13,14]. One characteristic of the CaO mold shell is that it is extremely easy to absorb the tide of water, which requires strict control of the humidity of the crucibles. Therefore, the control of the humidity increase the difficulty of the operation and hinder its application [15]. ZrO_2_ is thermodynamically stable and economical. However, a large number of inclusions were found in metals during the melting of a TiAl-based alloy with the ZrO_2_ crucible and this indicated that it had serious chemical reactions with the TiAl-based alloy at high temperature [16]. Al_2_O_3_ crucibles are used in the precision casting of TiAl-based alloys because of the high Al_2_O_3_ content in crucibles, which reduces the activity of TiAl-based alloys and their solid solubility to oxygen [17]. In addition, the thermal expansion coefficient of A1_2_O_3_ and TiAl-based alloys is very similar, which reduces the probability of fracture due to the low plasticity of TiAl-based alloys at room temperature. Therefore, the prospect of Al_2_O_3_ crucible in the casting of TiAl-based alloys is favored [18,19]. However, many studies have shown that the Al_2_O_3_ crucible also reacted with the melt during the melting process [20]. According to the study of Kostov and Frierich, Y_2_O_3_ presents the most thermodynamic stability among common metallic oxides, and it is a suitable material for crucibles used in the melting and casting of TiAl-based alloys [21]. Nevertheless, the poor thermal shock resistance of pure Y_2_O_3_ products and high production cost make it difficult to be widely used in industrial production [22].

Therefore, in order to find an appropriate crucible material, the composite crucibles were prepared by adding different amounts of Al_2_O_3_ powder and mixing different particle sizes of Y_2_O_3_. That can keep its stability during the smelting of high active TiAl-based alloys and make it cheap. The objective of this study was to demonstrate the mechanisms of the interactions between the chemical composition of Y_2_O_3_-Al_2_O_3_ composite crucibles and TiAl-based alloys, and then compare the stability of the composite crucibles with different Al_2_O_3_ contents during smelting.

## 2. Materials and Methods 

### 2.1. Preparation of Composite Crucibles

In this study, four different types of composite crucible with various components were designed and characterized. The compositions of the crucibles are listed in Table 1. The content of Y_2_O_3_ (purity 99.9%) (Beijing VPS-Tech Co., Ltd., Beijing, China) sand, which was 200 mesh and 60–80 mesh size of the main crystalline phase powder of the crucibles, was 30 wt % and 35 wt %, respectively. The content of 5 µm Al_2_O_3_ (purity 99.9%) (Beijing VPS-Tech Co., Ltd., Beijing, China) and 325 mesh Y_2_O_3_ active powder as the substrate of the crucible was 35 wt %. The compositions of the Al_2_O_3_-A, Al_2_O_3_-B, and Al_2_O_3_-C composite crucible with 5 µm Al_2_O_3_ powder instead of 325 mesh Y_2_O_3_ powder were 3.5 wt %, 7.0 wt % and 10.5 wt %, respectively. The crucibles were made of Y_2_O_3_ and Al_2_O_3_ powder by gelcasting with the dimensions of 25 mm o.d.× 18 mm i.d.× 30 mm length specified. For gelcasting, acrylamide [C_2_H_3_CONH_2_] (AM) (Beijing Lanyi Chemical Products Co., Ltd., Beijing, China) was used as a monomer, N,N′-methylenebisacrylamide [(C_2_H_3_CONH)_2_CH_2_] (MBAM) (Beijing Lanyi Chemical Products Co., Ltd., Beijing, China) as a coupling agent, N,N,N′,N′-tetramethylethylenediamine (TEMED) as a catalyst, ammonium persulphate (Beijing Chemical Works, Beijing, China) as an initiator and ammonium polyacrylate (Beijing Chemical Works, Beijing, China) as a dispersant. The crucible’s sintering temperature is 1873 K and the holding time is 4 h.

### 2.2. Melting Procedure

The master alloy, with nominal composition of Ti-47Al-2Cr-2Nb (at %), was prepared by an arc-melting method using Nb sheet (99.87%), commercial Ti sponge (99.76%), Al ingot (99.99%) and Cr granule (99.98%) as raw materials. The melting procedure was performed in a modified Bridgeman vacuum directional solidification furnace equipped with a removable composite crucible. Before each heating cycle, the chamber was evacuated down to ~10^−3^ Pa and backfilled with pure argon (O_2_ < 10 ppm; N_2_ < 50 ppm; H_2_ < 5 ppm; CH_4_ < 4 ppm) up to 0.05 MPa. The superheating temperature was 1873 K, measured and controlled by a thermocouple thermometer. The alloy ingots collected in composite crucibles were superheated for 10 min. Afterwards, the molten alloy was allowed to solidify in the composite crucible down to 300 K.

In order to further clarify the mechanism of interaction between molten metal and crucible, high temperature wetting experiments between TiAl-based alloys and crucibles were conducted by using composite ceramic substrates which were made under the same conditions of making composite crucibles. The oxide ceramic substrates with cylindrical shape were prepared by the dry pressing method using a uni-axial pressure machine (YLJ-40T, Shenyang Keji Automation Equipment Co., Ltd., Shenyang, China). Then, the Y_2_O_3_ and Al_2_O_3_ mixture powders were ground. Polyvinyl alcohol (PVA) (Beijing Lanyi Chemical Products Co., Ltd., Beijing, China) with a content of 5 wt % was added as a binder. The wetting experiment was carried out by the improved sessile drop equipment. Before heating, the furnace chamber was vacuumed to 9.9 × 10^−4^ Pa and further backfilled with a deeply purified Ar (99.999%) atmosphere to prevent active elements from evaporation and oxidation. When the temperature reached 1873 K, the alloy with the size of 3 × 3 × 3 mm^3^ in the top of the equipment was dropped. The spreading process was recorded by a CCD camera (Canon, EOS 80D, Tokyo, Japan). The contact angles were obtained by the ADSA-SESDROP and FTA32 software (Jilin University, Shenyang, China) [4,5]. 

After the melting experiment and wetting experiment, the microstructures of the alloys were examined by an electron probe micro-analyzer (EPMA, JXA-8100, JEOL, Tokyo, Japan), and the microstructures of crucibles were observed via scanning electron microscopy (SEM, JSM 6010, Japan Electronics Co., Ltd., Tokyo, Japan). Energy-dispersive spectrometry (EDS Oxford INCAPentaFET-x3, Japan Electronics Co., Ltd., Tokyo, Japan) was used to analyse the chemical composition and impurity elements at specified positions from the surface to the inside of the ingots to establish the homogeneity. The phase identification of the compounds on the alloy matrix and contact interface was performed by X-ray diffraction (XRD, D/max 2200PC, Rigaku, Tokyo, Japan) with Cu Kα radiation by scanning on the designated area.

## 3. Results

### 3.1. Microstructure

**Figure 1:**
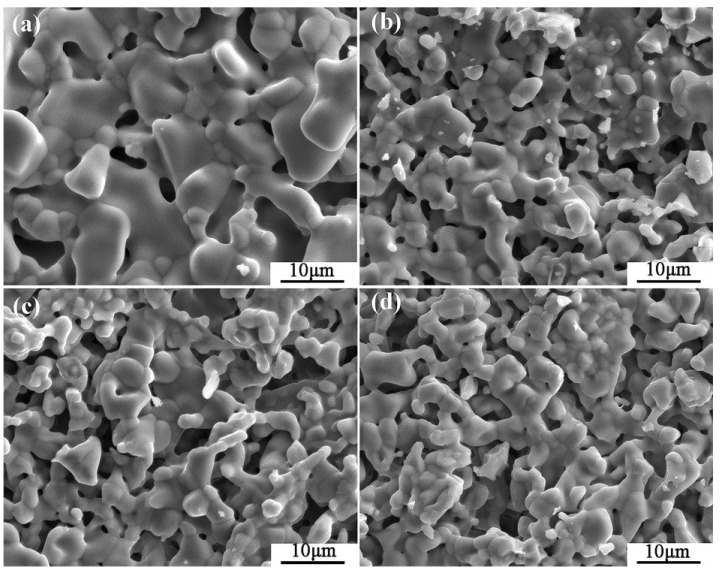
The SEM micrographs of the composite crucibles with specific components. (**a**) The pure Y_2_O_3_ crucible; (**b**) Al_2_O_3_-A crucible, containing 3.5 wt % Al_2_O_3_; (**c**) Al_2_O_3_-B crucible, containing 7.0 wt % Al_2_O_3_; (**d**) Al_2_O_3_-C crucible, containing 10.5 wt % Al_2_O_3_.

**Figure 2:**
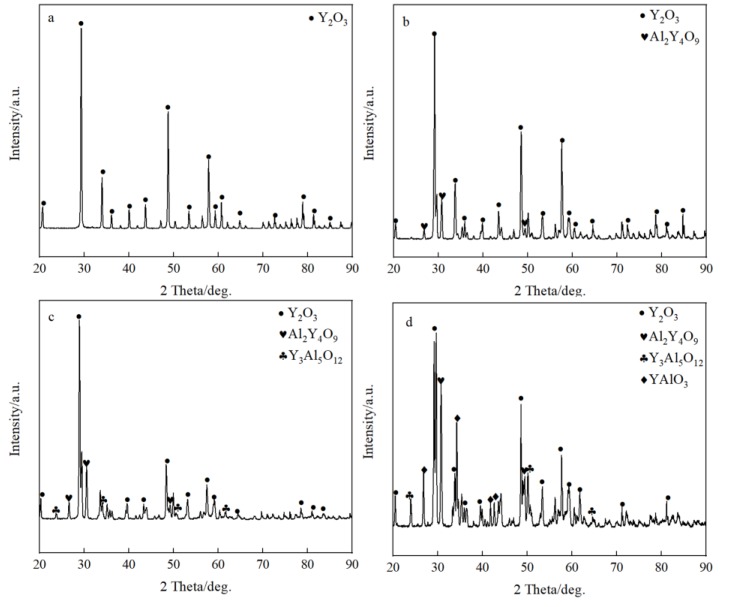
**T**he XRD spectrum of composite crucibles before the melting experiment. (**a**) The pure Y_2_O_3_ crucible; (**b**) Al_2_O_3_-A crucible, containing 3.5 wt % Al_2_O_3_; (**c**) Al_2_O_3_-B crucible, containing 7.0 wt % Al_2_O_3_; (**d**) Al_2_O_3_-C crucible, containing 10.5 wt % Al_2_O_3_.

The typical microstructures of four composite crucibles after sintering are shown in Figure 1. The corresponding porosities of the composite crucibles are shown in Table 2. The morphology of the pure Y_2_O_3_ crucible (Figure 1a), which was a pure Y_2_O_3_ crucible without addition of Al_2_O_3_, presented the least amount of connected pores and the densest surface. Compared with several other crucibles, it was the most thoroughly sintered. The degree of sintering of the Al_2_O_3_-A crucible was less than that of the pure Y_2_O_3_ crucible. The number of surface connected pores increased and the porosity increased from 5.88% to 9.93%. The surface microstructures of the Al_2_O_3_-B and Al_2_O_3_-C crucibles were very similar, with similar porosity values of 11.52% and 11.34%, respectively. Compared to the Al_2_O_3_-A crucible, the number of surface connected pores increased significantly, with more even dispersal, and the combination of oxide particles was looser. The XRD spectrum (Figure 2) shows that there was only Y_2_O_3_ in the pure Y_2_O_3_ crucible, and Al_2_O_3_-A crucible contained the Al_2_Y_4_O_9_ phase. In addition to the Al_2_Y_4_O_9_, Y_3_Al_5_O_12_ and YAlO_3_ phases were newly formed in the Al_2_O_3_-B and Al_2_O_3_-C crucible with higher Al_2_O_3_ addition.

### 3.2. Melt-Crucible Interface

**Figure 3:**
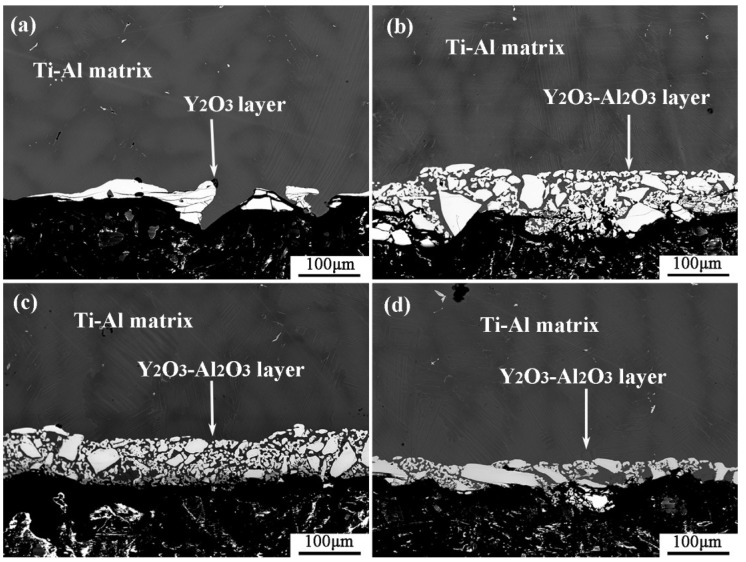
Microstructure of the melt-crucible contact interface. (**a**–**d**) represent the pure Y_2_O_3_, Al_2_O_3_-A, Al_2_O_3_-B and Al_2_O_3_-C crucibles, respectively.

**Figure 4:**
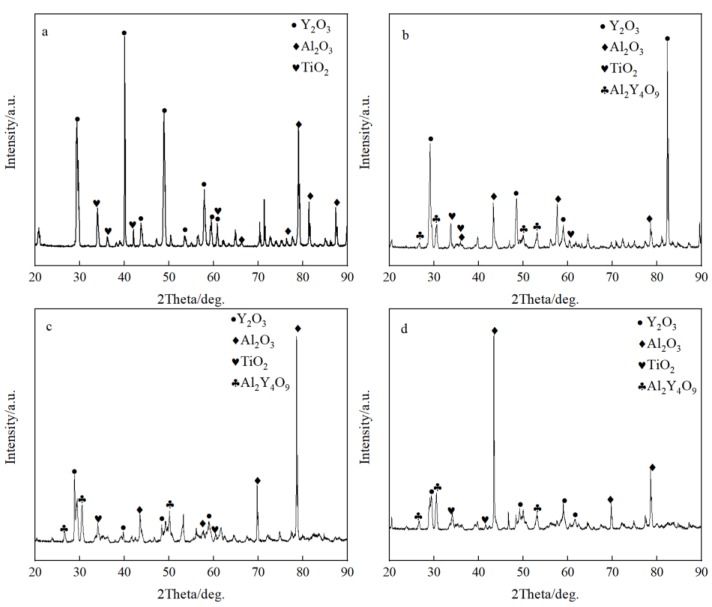
The XRD spectrum of the interface. (**a**–**d**) represent the pure Y_2_O_3_, Al_2_O_3_-A, Al_2_O_3_-B and Al_2_O_3_-C crucibles, respectively.

Figure 3 shows the microstructure of the metal-crucible contact interface after the melting experiment. The molten metal penetrated into the crucible at the interface and then adhered to oxide layer from the crucible. As shown in Figure 3a, the interface layer in the pure Y_2_O_3_ crucible was discontinuous. The greatest thickness was about 100 µm, while the thinnest was about only a few microns. It can be seen that the white substance bonded to the metal was almost a complete Y_2_O_3_ coarse sand particle. The interface layers of Al_2_O_3_-A, Al_2_O_3_-B and Al_2_O_3_-C crucible (Figure 3b–d) were continuous and uniform. Compared with the pure Y_2_O_3_ crucible, the oxide particles that adhered to the metal were relatively small. The thickness of the interface layer of the Al_2_O_3_-A crucible was the largest, about 150 µm, and the particles in the interface layer were fine and loose. The thickness of the interface layer of Al_2_O_3_-B crucible was about 100 µm, and the white particles were fine, but the arrangement was relatively close relative to that of the Al_2_O_3_-A crucible. The thickness of the interface layer of the Al_2_O_3_-C with the most Al_2_O_3_ powder was about 50 µm, and the proportion of fine white oxide particles decreased. The XRD diffraction pattern of the interface layer (Figure 4) showed that the interface layer of the pure Y_2_O_3_ crucible had Y_2_O_3_, Al_2_O_3_ and a small amount of TiO_2_, but at the interface of Al_2_O_3_-A, Al_2_O_3_-B and Al_2_O_3_-C crucibles, besides the above three substances, Al_2_Y_4_O_9_ was also detected.

**Figure 5:**
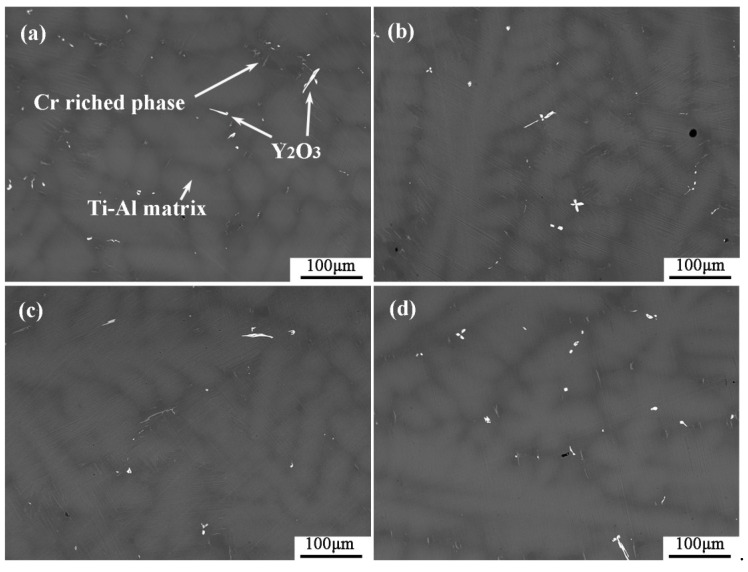
(**a**–**d**) are the internal microstructures of the metal particles obtained from the alloy melting experiments of pure Y_2_O_3_, Al_2_O_3_-A, Al_2_O_3_-B and Al_2_O_3_-C crucibles.

**Figure 6:**
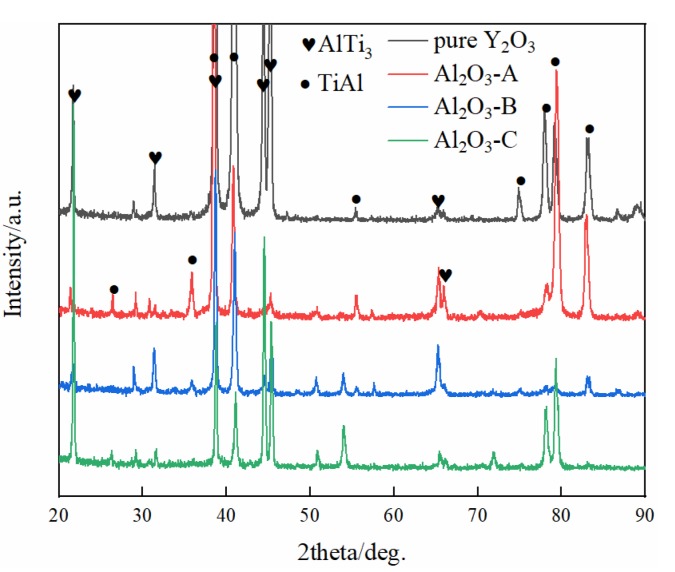
The XRD spectra of the interior of metal particles.

Figure 5 shows the microstructures of the interior of the alloys after the melting experiment. A large number of coarse grayish dendrites were distributed in the dark gray matrix, and a small number of irregular phases with bright white and grey contrast were dispersedly distributed among them. The grey precipitates were mainly distributed in the deep gray between dendrites, presented as tiny needles. Bright white precipitates were mainly distributed among dendrites, and there were a small number of dendrites. Some of them were elongated, some were plum-like flowers, and some were distributed small particles. The XRD spectra of the interior of metal particles after melting experiments are shown in Figure 6. There were only two phases of TiAl and AlTi_3_. The presumed results may not be shown in diffraction patterns due to too little precipitation. The EDS analysis of the precipitated bright with white contrast and gray matter contrast is shown in Table 3. The atomic percentage of heavy Cr in the gray contrast reached 23–25%, and it is known that it was a rich Cr phase precipitate, and the Cr in the matrix was easily segregated in the interdendritic area. The ratio of Y and O in white contrast material was close to 1:1, and at the same time, contained a small amount of Al. It is speculated that it was an oxide that contained Y and Al, and it was formed during the melting process of the metal from the crucible to the metal melt.

**Figure 7:**
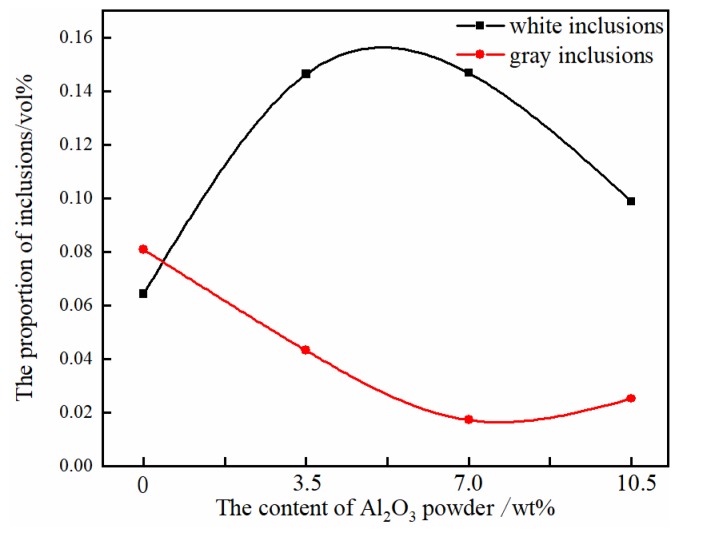
The number of precipitates in metal particles.

The percentage of the gray interlining Cr rich and the white interlining yttrium aluminum oxide in the alloy interface area was calculated, which was used to indicate the content of inclusions in the TiAl alloy. The results showed that (Figure 7) with the increase of the addition of Al_2_O_3_ powder, the white inclusion content in the metal increased first and then decreased. The content of white inclusions in the pure Y_2_O_3_ crucible with no Al_2_O_3_ powder was the lowest. After adding a small amount of Al_2_O_3_ powder, the number of inclusions increased rapidly. With the increase of the addition of Al_2_O_3_, the content of white inclusions decreased. After the addition of Al_2_O_3_, the content of the rich Cr phase of the gray contrast decreased first, followed by a rebound trend, and the content of the precipitated phase was the lowest when the proportion of Al_2_O_3_ powder was 7.0 wt %.

## 4. Discussion

The surface microstructure and the porosity of the crucibles showed that the addition of Al_2_O_3_ affected the degree of sintering of the crucibles. As shown in Figure 1 and Table 2, the sintering degree of Y_2_O_3_ crucible decreased with the addition of Al_2_O_3_, and the porosity increased with the addition of Al_2_O_3_ before maintaining a relatively stable value. According to the results obtained by H.R. Zhang [23], Y_2_O_3_ and Al_2_O_3_ reacted in the process of high-temperature sintering to form dense and solid Y_3_Al_5_O_12_, which increased the sintering degree of the crucibles. In this study, the XRD (Figure 2) detected Al_2_Y_4_O_9_, Y_3_Al_5_O_12_ and YAlO_3_ phases in the composite crucibles with Al_2_O_3_ added before the melting experiment. The system Y_2_O_3_-Al_2_O_3_ features Al_2_Y_4_O_9_, YAlO_3_ and Y_3_Al_5_O_12_, with mole ratios Y_2_O_3_: Al_2_O_3_ of 2:1, 1:1, and 3:5, respectively. The reaction temperature and ratio of Y_2_O_3_ and Al_2_O_3_ affect the type of final product [24]. Kolitsch, U. et al. suggested that YAlO_3_ is a stable phase in the temperature range from 1873 K to 1673 K and possibly down to ambient temperature [25]. However, a reversible phase transition of Al_2_Y_4_O_9_ at around 1650 K has been reported [26]. The reactions between Y_2_O_3_ and Al_2_O_3_ produced several different products as described above during the sintering process because Y_2_O_3_-Al_2_O_3_ is a rather complex (and partly metastable) system. The Al_2_O_3_ powder with size of 5 µm was added to the Al_2_O_3_-A, Al_2_O_3_-B and Al_2_O_3_-C crucibles, and an alternative part of the 325 mesh Y_2_O_3_ powder formed the mixed fine powder. Because the particle size of Al_2_O_3_ powder was much smaller than that of the 325 mesh Y_2_O_3_ powder, and the 325 mesh Y_2_O_3_ of 3.5 wt % in the Al_2_O_3_-A crucible was replaced by Al_2_O_3_ powder of 5 µm, and the surface grain of the small crucible formed by sintering was finer than that on the surface of the pure Y_2_O_3_ crucible. The proportion of Al_2_O_3_ powder in Al_2_O_3_-B and Al_2_O_3_-C crucibles continued to increase. The particles were in close contact with each other, and the sintering degree was good. Therefore, compared to the Al_2_O_3_-A crucible, the porosity of the Al_2_O_3_-B and Al_2_O_3_-C crucible was only slightly increased, and the surface micromorphology of the two was similar.

The interfacial reactions between melt metals and ceramic crucibles are very complicated physical-chemical process. The experimental results in this study showed that the alloy melting experiments were carried out in the crucibles with different compositions under the same experimental conditions, and the amount of oxide inclusions in the metal was different after the experiment. The amount of oxide inclusions in the alloy melted with pure Y_2_O_3_ crucible was the smallest. However, in the composite crucibles, the amount of oxide inclusions decreased with the addition of Al_2_O_3_. When the amount of Al_2_O_3_ added was 3.5 wt %, the proportion of inclusions in the metal was largest.

**Figure 8:**
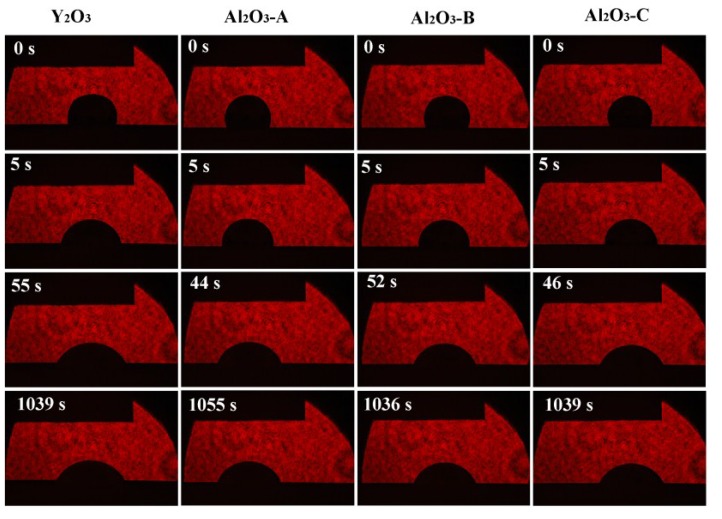
Wetting process of the Ti-47Al-2Cr-2Nb alloys on substrates with various compositions at 1873 K.

**Figure 9:**
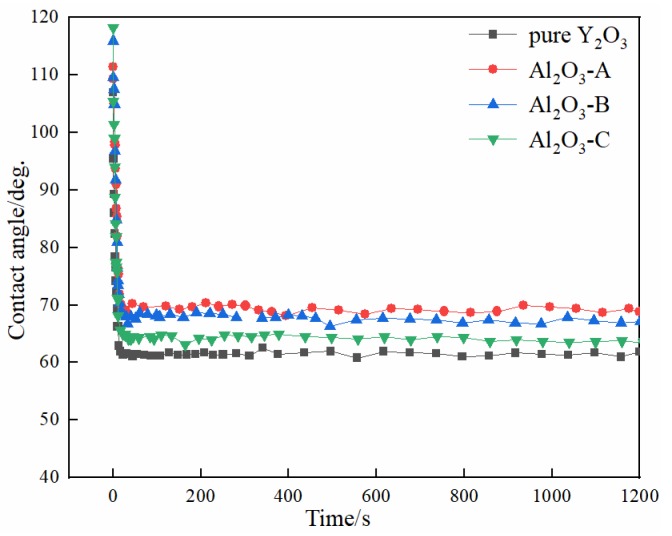
Variation of contact angle with time at 1873 K.

From the chemical point of view, the pure Y_2_O_3_ crucible without Al_2_O_3_ was more thermodynamically stable than composite crucibles with Al_2_O_3_. The Al_2_O_3_ and TiO_2_ detected in the interfacial layer (Figure 4) were presumably speculated to be formed by the combination of free O and Ti atoms and Al atoms in the melt during the metal melting experiment. In the metal melting experiment, the amount of oxide inclusions in the pure Y_2_O_3_ crucible entering the metal was the least because Y_2_O_3_ has higher chemical stability than Al_2_O_3_ and is less likely to react chemically with the molten alloy. Therefore, the number of oxide inclusions in the alloy obtained by Al_2_O_3_-A, Al_2_O_3_-B and Al_2_O_3_-C crucibles was higher than that of the pure Y_2_O_3_ crucible. Secondly, from the physical effect, the high temperature metal melt has a physical erosion effect on the crucible. The microstructure of the crucible surface (Figure 1) showed that the Y_2_O_3_ particles were the largest on the surface of the pure Y_2_O_3_ crucible, the porosity was the smallest and the surface was the most compact. In order to further clarify the mechanism of interaction between molten metal and the crucible, high temperature wetting experiments between metal and crucibles were performed. As shown in Figure 8 and Figure 9, the initial contact angles and the equilibrium contact angles of the molten metal and crucible varied with the change in composition. The initial contact angles of the pure Y_2_O_3_, Al_2_O_3_-A, Al_2_O_3_-B and Al_2_O_3_-C were 107°, 111.4°, 115.9° and 118.2°, respectively. The equilibrium contact angles were 61.5°, 69.3°, 67.7° and 64.2°, respectively. During the melting experiment, the high temperature metal melt contacted well with the crucible, and the crucible itself had high strength. Liquid metal can quickly form a protective film on the surface of the crucible, thus preventing more oxide particles from the crucible falling into the metal melt. The Al_2_O_3_-C crucible surface microstructure had fine grains, and the highest content of Al_2_O_3_ added to produce more Y_3_Al_5_O_12_. Y_3_Al_5_O_12_ belongs to cubic crystal system, with garnet structure, high temperature resistance and strength at high temperatures, and its wettability was similar to that of the pure Y_2_O_3_ crucible, so it can reduce the loss to the oxide crucible alloy. The degree of interaction between the melt and composite crucible (Al_2_O_3_-A and Al_2_O_3_-B) is stronger than that between the pure Y_2_O_3_ crucible and the melt, and weaker than that between the Al_2_O_3_-C crucible and the melt. The crucible structure was relatively loose, and the wettability between metal and interface was relatively poor. Therefore, the corrosion of metal in the crucible was considerable, and the content of oxide inclusions in molten alloys was higher than that in pure Y_2_O_3_ and Al_2_O_3_-C.

## 5. Conclusions

The TiAl-based alloy was melted in Y_2_O_3_-based composite crucibles with an Al_2_O_3_ content of 0 wt %, 3.5 wt %, 7.0 wt % and 10.5 wt %, based on which the interfacial reactions and associated mechanisms between TiAl-based alloy and composite crucibles were discussed. The primary conclusions of this work were as follows.

The chemical stability of the pure Y_2_O_3_ crucible was the best. After the melting experiment, the number of inclusions in the interior of the metal particles and the reactants at the interface layer with the pure yttrium crucible were the lowest. With the increase in the amount of Al_2_O_3,_ the interfacial reaction between the composite crucible and the molten alloy became weaker. When the content of Al_2_O_3_ in the composite crucible increased from 3.5 wt % to 10.5 wt %, the thickness of the interface layer of the melt-crucible decreased from 150 µm to 50 µm. The addition of Al_2_O_3_ increased the wettability of the composite crucible with the metal melt. The equilibrium contact angles between metal and crucible under 1873 K decreased from 69.3° to 64.2°.

## Figures and Tables

**Table 1:** Designated composition of composite crucibles with different Al_2_O_3_ contents.

Crucible	5 μm Al_2_O_3_ Powder/(g)	325 mesh Y_2_O_3_ Powder/(g)	200 mesh Y_2_O_3_ Sand/(g)	60–80 mesh Y_2_O_3_ Sand/(g)
Pure Y_2_O_3_	0	32.50	27.85	32.50
Al_2_O_3_-A	3.17	28.53	27.85	32.50
Al_2_O_3_-B	6.19	24.76	27.85	32.50
Al_2_O_3_-C	9.07	21.16	27.85	32.50

**Table 2:** The porosity of crucibles.

Crucible	Pure Y_2_O_3_	Al_2_O_3_-A	Al_2_O_3_-B	Al_2_O_3_-C
Porosity	5.88%	9.93%	11.52%	11.34%

**Table 3:** The internal components of the metal particles.

Phases	Compositions					
	Al	Ti	Cr	Nb	Y	O
Dark gray grain boundary	50.37	43.84	4.5	1.29	-	-
Light gray dendrite	43.30	52.73	1.33	2.64	-	-
Light gray inclusions	34.92	40.55	23.40	1.13	-	-
	32.54	41.67	24.91	0.89	-	-
Bright white inclusions	1.59	2.77	-	-	49.09	46.55
	1.28	2.41	-	-	49.51	46.8
